# Flavonoids in MASLD: preclinical mechanisms, pharmacological targets, and translational challenges

**DOI:** 10.3389/fphar.2026.1878277

**Published:** 2026-07-24

**Authors:** Zhonghai Dao, Jishen Li, Yiping Liu, Deqiong Niu, Zhifeng Xiao, Jinrun Dong

**Affiliations:** 1 College of Biological Science and Food Engineering, Southwest Forestry University, Yunnan, China; 2 People’s Hospital of Fengqing County, Yunnan, China

**Keywords:** cellular and animal models, challenges and opportunities, clinical trial, flavonoids, metabolic dysfunction-associated steatotic liver disease (MASLD)

## Abstract

Metabolic dysfunction-associated steatotic liver disease (MASLD) has become the most prevalent chronic liver disease worldwide, yet no pharmacological therapy has achieved regulatory approval. Flavonoids, plant-derived polyphenols encompassing seven structural subclasses, exhibit considerable preclinical promise through multi-target mechanisms but face translational barriers owing to poor oral bioavailability and insufficient clinical validation. This review systematically evaluates 33 structurally characterized single flavonoids for their therapeutic mechanisms, pharmacological targets, and translational prospects in MASLD, integrating evidence from cellular models, diverse rodent models, and available clinical trials. A tiered evidence classification (Levels A–C) was applied based on clinical data availability, multi-model validation, mechanistic depth, and study design rigor. Mechanistically, flavonoids restore hepatic lipid homeostasis by concurrently inhibiting SREBP-1c-mediated *de novo* lipogenesis and promoting PPARα-driven fatty acid β-oxidation via AMPK activation; ameliorate insulin resistance through IRS-1/PI3K/Akt signaling; attenuate hepatic inflammation by suppressing NF-κB/NLRP3 inflammasome cascades; reinforce antioxidant defenses via Nrf2/ARE-mediated induction of HO-1, SOD, and GPX4 with concomitant ferroptosis inhibition; enhance autophagic-lysosomal lipid clearance through TFEB nuclear translocation and Sirt1-dependent lipophagy; and remodel gut microbiota composition to fortify intestinal barrier integrity. Genistein, dihydromyricetin, quercetin, and kaempferol exemplify polypharmacological engagement across multiple pathways. Despite robust mechanistic evidence, oral bioavailability remains limited to 1%–5% owing to poor aqueous solubility, extensive phase II conjugation, and food-matrix interactions. Emerging strategies—carbamate prodrugs, nanoliposomes, biomimetic nanoemulsions, and colon-targeted nanoparticles—demonstrate feasibility in surmounting these barriers. Clinical evidence reveals compound-specific efficacy profiles: hesperidin reduces steatosis and transaminases; genistein improves insulin sensitivity; naringenin ameliorates lipid profiles without altering fibrosis markers. Critical appraisal identifies persistent limitations including small sample sizes, predominant reliance on male animals, short intervention durations, and absence of biopsy-confirmed endpoints. Future research must prioritize rigorous multicenter randomized controlled trials with optimized formulations, comparative efficacy studies, systematic safety evaluations, and multi-omics integration to bridge the translational gap toward evidence-based flavonoid therapeutics for MASLD.

## 1 Introduction

Nonalcoholic fatty liver disease (NAFLD) is the most prevalent chronic liver disease globally. Its pathological spectrum mainly comprises non-alcoholic fatty liver (NAFL) and non-alcoholic steatohepatitis (NASH). Advanced NAFLD may progress to complications such as liver cirrhosis and hepatocellular carcinoma (HCC) (Loomba et al., 2021; Wei et al., 2024). NAFLD already affects approximately 38% of the adult population globally and has evolved into a major public health challenge (Xu et al., 2024). In China the prevalence of NAFLD has grown significantly over the past 2 decades from 1999 to 2018, from 23.8% in the early 2000s to 32.9% in 2018, with more than 240 million people living with NAFLD, accounting for more than one-fifth of the global NAFLD population (Zhou et al., 2020).

NAFLD is considered to be the hepatic manifestation of the metabolic syndrome and is usually closely associated with metabolism-related risk factors such as obesity, dyslipidemia, hypertension and diabetes mellitus (Wei et al., 2024). The pathogenesis of NAFLD is complex, with the original “two-hit hypothesis” in which the first strike is caused by the accumulation of fat in the liver, resulting from factors such as poor lifestyle and insulin resistance, and the second strike is that the liver becomes more sensitive to further damage, leading to inflammation and fibrosis (Buzzetti et al., 2016). However, this view was quickly recognized as an oversimplification that did not fully encapsulate the complexity of NAFLD, as the synergistic effects of multiple parallel factors in genetically susceptible individuals are involved in disease progression, as suggested by the “multiple parallel strikes” theory, which suggests that NAFLD is caused by a combination of metabolic disorders, insulin resistance, hepatic fat accumulation, chronic inflammation, oxidative stress, endoplasmic reticulum stress, gut dysbiosis, genetic factors, and lifestyle (Tilg et al., 2021; Guo et al., 2022; Wang L. et al., 2023). These factors may act individually or in combination on the liver, leading to the accumulation of fat within the liver cells (hepatic steatosis) and may further progress to non-alcoholic steatohepatitis (NASH) and even cirrhosis and hepatocellular carcinoma (HCC). Given the evolving knowledge of NAFLD pathogenesis, a new nomenclature system has been adopted: NAFLD is referred to as MASLD, and NASH is redefined as MASH (Rinella et al., 2023; Li Y. et al., 2024). Early pharmacologic intervention is essential for the treatment of MASLD. Early-stage MASLD is generally asymptomatic. Progression to fibrotic MASLD can ultimately lead to liver cirrhosis. where not only the disease progression worsens significantly, but also the effectiveness of clinical medications is significantly reduced (Cao Y. et al., 2023). Current treatment for MASLD focuses on lifestyle improvements, dietary modifications, and the use of bariatric surgery, but MASLD patients often have difficulty maintaining an improved lifestyle, and lifestyle changes alone are often insufficient to reach the weight thresholds needed to significantly reverse nonalcoholic steatohepatitis (MASH) and avoid cirrhosis, nevertheless, there is still no clinically recognized agent for the treatment of MASLD (Brunt et al., 2015; Guo et al., 2022; Genua and Cusi, 2024). Therefore, it is of great practical significance to strengthen the research on the pathogenesis of MASLD and to search for safe and effective drugs for the prevention and treatment of MASLD. Alongside in-depth investigations into the pathological mechanisms of MASLD, growing efforts have been devoted to screening effective natural products and their extracts, which in turn propels the ongoing development of targeted therapeutic drugs.

**FIGURE 1:**
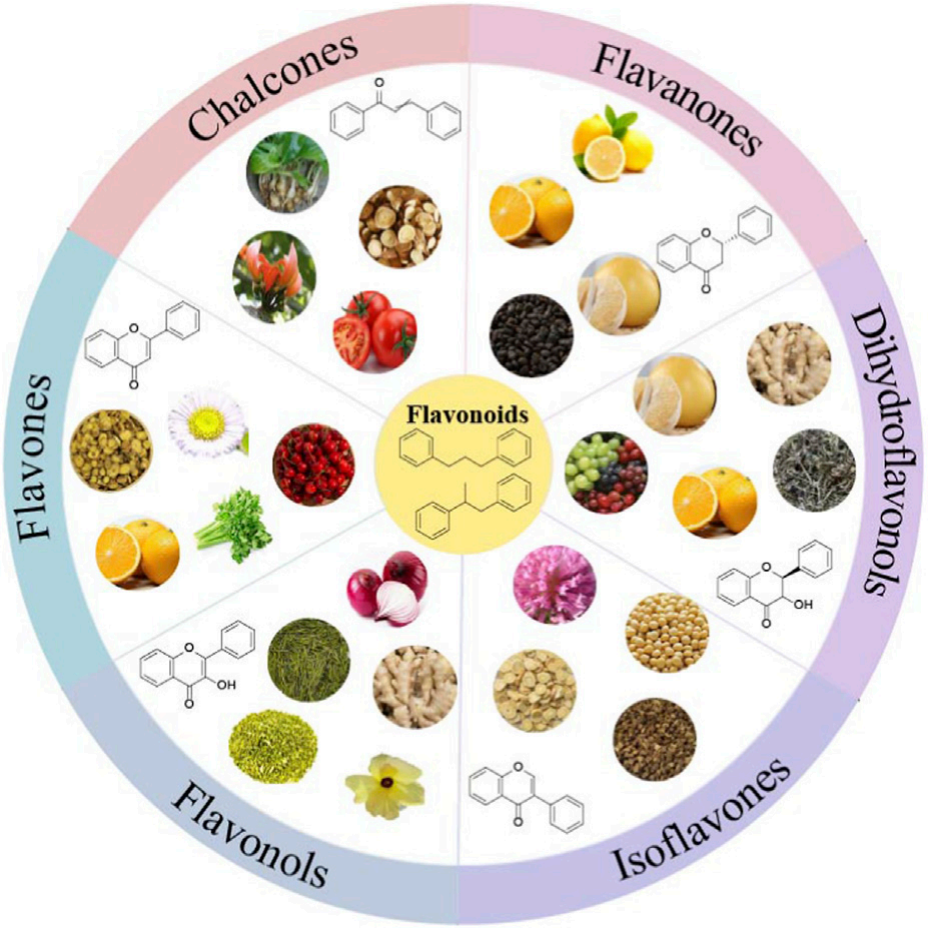
Classification and sources of common flavonoids.

**FIGURE 2:**
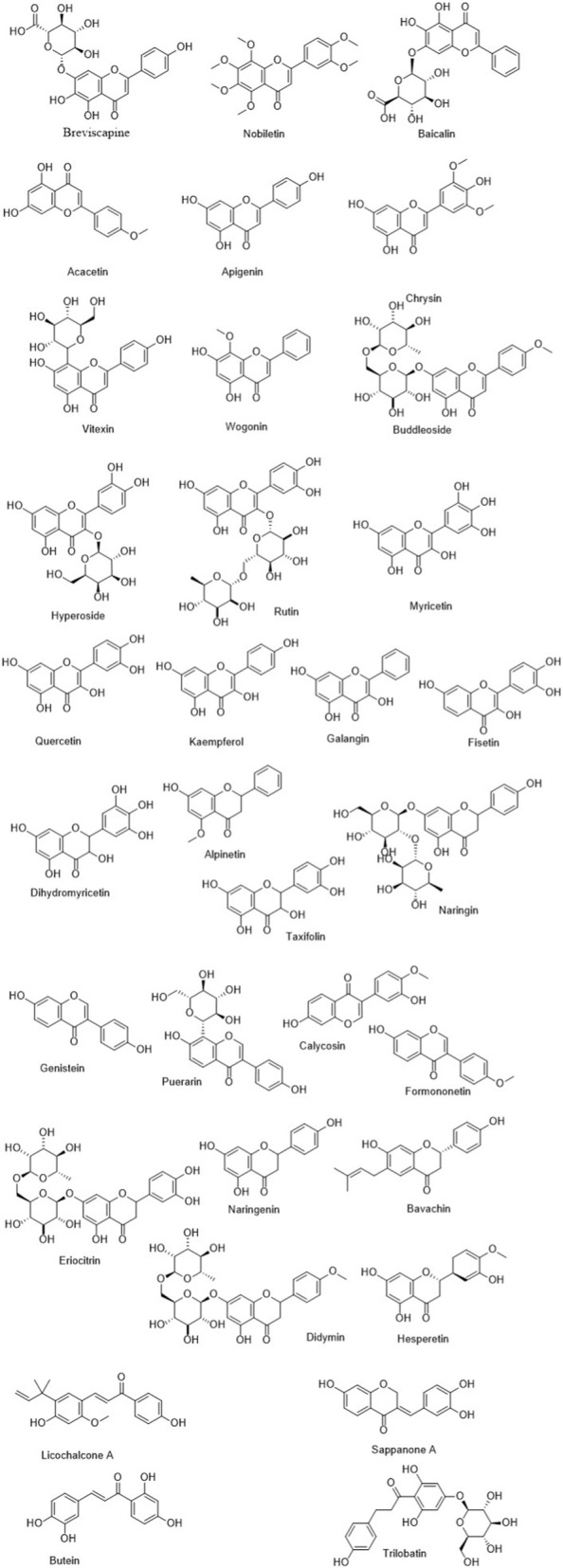
Basic skeletal structures of 33 flavonoids.

Flavonoids are a class of important natural products, belonging to a class of plant secondary metabolites with polyphenolic structure, widely present in fruits, vegetables and many parts of plants (Panche et al., 2016). According to the position of their benzene ring substituents and the degree of oxidation, flavonoids can be divided into several subclasses such as flavonoids, flavonols, flavanones, isoflavones, flavanols, chalcones, and anthocyanins, and the representative compounds include quercetin (flavonol), apigenin (flavonol), naringenin (flavanone), and genistein (isoflavonoid), etc., (Figure 1) (Okoye et al., 2024). In recent years, studies have confirmed that flavonoids exhibit unique advantages in nutritional healthcare and disease intervention through their multidimensional pharmacological mechanisms. These advantages stem from their antioxidant, anti-inflammatory, anti-mutagenic, and anticancer properties, as well as their ability to modulate key cellular enzyme functions (Tan et al., 2022). These natural products precisely cover the core pathology of MASLD by regulating the dynamic balance of metabolism related to lipid synthesis and catabolism, improving insulin signaling, inhibiting pro-inflammatory factor cascade, and enhancing the antioxidant defense system, among other multidimensional effects (Serafini et al., 2010; Vissenaekens et al., 2022). This therapeutic property based on multi-target synergy makes flavonoids a focus of research for novel therapeutic strategies for MASLD. In this review, we summarized the therapeutic mechanisms through which 33 flavonoid single compounds (Figure 2) target MASLD and provided insights to guide future research aimed at identifying novel drug candidates for MASLD treatment.

## 2 Methods

To systematically review relevant research from the past decade (2015–2025), we conducted a comprehensive literature search in databases such as Web of Science, PubMed, and ScienceDirect. The search employed a strategy combining Medical Subject Headings (MeSH) terms with free-text keywords to maximize sensitivity. Specific keywords and their combinations included: NAFLD, MASLD, NASH, MASH, flavonoids, lipid metabolism, hepatic lipid accumulation, oxidative stress, inflammation, and insulin resistance.

After deduplication, a preliminary screening was conducted based on titles and abstracts, followed by a full-text review of articles that potentially met the inclusion criteria. Additionally, the reference lists of selected reviews and meta-analyses were manually searched to identify any studies that may have been overlooked in the database search. Final inclusion depended not only on the relevance of the study topic but also on a rigorous evaluation of the quality of preclinical experiments: We focused on whether studies employed multiple complementary model systems (such as a combination of animal models from different species and various cell lines) for cross-validation; whether the sample size was statistically sound; whether the duration of pharmacological intervention was sufficient to evaluate chronic regulatory effects on hepatic lipid metabolism; and whether appropriate positive controls (such as approved drugs or well-established model inducers/inhibitors) were included. Based on the data extracted above, we will establish a tiered evidence classification or strength ranking for each flavonoid compound and present this in a summary table, addressing factors such as: whether clinical evidence exists, whether the findings have been validated across multiple models, or whether in-depth mechanistic investigations have been conducted using genetic or pharmacological approaches.

To help readers distinguish well-validated findings from preliminary observations, we assigned each compound to one of four evidence tiers based on three criteria: (i) whether the finding has been tested in animal models, cell models, or both; (ii) whether the proposed molecular target has been validated by gene knockout or specific pharmacological inhibitor (causality) rather than mere expression-level correlation; and (iii) whether any clinical data exist. The tiers are defined as follows:

Grade A (Clinical evidence available): At least one randomized controlled trial in MASLD patients, plus animal studies with target validation by gene knockout or specific inhibitor.

Grade B (Strong preclinical, target validated): At least two animal models plus cell models; the core mechanism has been confirmed by gene knockout/knockdown *in vivo* or specific pharmacological inhibitor reversal. No clinical data yet.

Grade C (Moderate preclinical, correlative): At least one animal model plus cell models; mechanism inferred from changes in pathway protein/mRNA levels, but without gene knockout or specific inhibitor confirmation.

Grade D (Preliminary): Cell models only, or a single animal study without independent replication; or *in silico* prediction without experimental validation.

## 3 Multiple hits of MASLD

### 3.1 Energy metabolism

The liver serves as a critical hub for numerous physiological processes, including macronutrient metabolism, blood volume regulation, immune system support, endocrine control of growth signaling pathways, lipid and cholesterol homeostasis, and catabolism of xenobiotic compounds, including many existing drugs (Trefts et al., 2017). In MASLD, disturbances in hepatic lipid metabolism trigger excessive fat deposition in the liver. As energy metabolic dysfunction serves as a core driver of MASLD pathogenesis and progression, systematic analysis of key molecular pathways and regulatory networks is crucial for clarifying disease mechanisms, discovering intervention targets, and advancing precise and effective therapeutic strategies.

Sterol regulatory element binding protein 1c (SREBP-1c) is a key transcription factor predominantly expressed in tissues such as the liver, white adipose tissue, and skeletal muscle. SREBP-1c plays a central role in regulating lipid metabolism, primarily by activating the expression of genes involved in fatty acid synthesis. According to studies, SREBP-1c specifically activates the transcription of key lipid synthases such as fatty acid synthase (FASN) and acetyl coenzyme A carboxylase (ACC). In NAFLD, aberrant activation of SREBP-1c is associated with/contributes to the upregulation of its downstream target genes. FASN, a pivotal enzyme in *de novo* fatty acid synthesis, acts synergistically with ACC, which catalyzes the rate-limiting step of fatty acid biosynthesis. Synergistic upregulation of their expression levels culminates in intracellular lipid accretion in hepatic parenchymal cells. This metabolic disorder plays an important role in the pathogenesis of MASLD and serves as a key molecular basis for the progression of hepatocellular steatosis to inflammatory and fibrotic stages (DeBose-Boyd and Ye, 2018; Li N. et al., 2023).

PPARα (peroxisome proliferator-activated receptor alpha), a member of the nuclear receptor superfamily, is mainly localized and expressed in highly metabolically active tissues such as the liver, kidney, cardiac muscle and skeletal muscle (Lefebvre et al., 2006). This receptor plays a central role in the pathophysiological mechanisms of nonalcoholic fatty liver disease (MASLD) by modulating the transcriptional activity of key genes for fatty acid metabolism. Ligand activation of PPARα ameliorates hepatic fatty acid metabolic dysregulation in MASLD through dual synergistic mechanisms: it enhances mitochondrial and peroxisomal β-oxidation by transcriptionally upregulating rate-limiting enzymes (notably carnitine palmitoyl transferase 1 [CPT1] and acyl-CoA oxidase 1 [ACOX1]), while concurrently augmenting hepatocellular uptake of circulating fatty acids via induction of the solute carrier transporter SLC27A1 (FATP-1) and the scavenger receptor CD36 (Fougerat et al., 2020). In the hepatic microenvironment, the above metabolic regulatory network mediated by PPARα significantly reduces the abnormal accumulation of free fatty acids in hepatocytes, thereby effectively alleviating the pathological process of hepatocellular steatosis. Furthermore, PPARα regulates ketogenesis, converting acetyl-CoA derived from β-oxidation into ketone bodies. This process provides alternative energy substrates for peripheral tissues and represents a physiologically significant compensatory mechanism for maintaining systemic energy homeostasis (Pawlak et al., 2015; Chen et al., 2023).

Adenosine monophosphate-activated protein kinase (AMPK) is a core regulator of cellular energy metabolism and is known as the “guardian of cellular energy”. It is activated during energetic stress by sensing changes in the intracellular AMP/ATP ratio and rapidly initiates a series of metabolic reprogramming reactions. The main activation site of AMPK is Thr172 on the α-subunit, and its phosphorylation is mediated by the upstream kinases LKB1 or CAMKK2, facilitated by AMP- or ADP-binding to the γ-subunit, and simultaneously inhibited by ATP (Garcia and Shaw, 2017). Activated AMPK inhibits ATP-consuming anabolic pathways, including hepatic gluconeogenesis, fatty acid synthesis, and cholesterol biosynthesis and activates ATP-generating catabolism (e.g., fatty acid oxidation, glycolysis, autophagy, and mitochondrial biosynthesis) by phosphorylating downstream target proteins (Herzig and Shaw, 2018). Thus, AMPK plays a key role in maintaining energy homeostasis, regulating lipid and glucose metabolism, and promoting mitochondrial quality control (Carling, 2017). Due to its central position in metabolic regulation, AMPK has become an essential target for studying the mechanisms of metabolic diseases (e.g., Diabetes, obesity, MASLD.) and developing therapeutic strategies.

### 3.2 Insulin resistance

Insulin resistance contributes to the development and progression of MASLD through a variety of mechanisms, including increased adipose tissue inflammation, elevated serum free fatty acid levels, increased hepatic lipogenesis, intestinal flora dysbiosis, and mitochondrial dysfunction. Adipose tissue inflammation promotes the release of pro-inflammatory cytokines, such as tumor necrosis factor alpha (TNF-α) and interleukin 6 (IL-6). These cytokines, in turn, exacerbate insulin resistance by activating various protein kinases and inhibiting insulin signaling pathways (Hotamisligil et al., 1993). Meanwhile, patients with insulin resistance exhibit elevated serum levels of free fatty acids (FFAs). These FFAs are taken up by the liver and converted into triacylglycerols. Importantly, metabolic intermediates generated during this process, such as diacylglycerols (DAGs) and ceramides, can activate protein kinases like protein kinase C (PKC). This activation further inhibits the insulin signaling pathway, thereby forming a vicious cycle. In addition, insulin resistance promotes hepatic lipogenesis by regulating transcription factors such as sterol regulatory element binding protein 1c (SREBP-1c) and carbohydrate response element binding protein (ChREBP) (Benhamed et al., 2012). Furthermore, intestinal dysbiosis can induce an inflammatory response that affects fat distribution and insulin sensitivity, which in turn exacerbates the pathologic process of MASLD. Mitochondrial dysfunction is closely associated with impaired fatty acid β-oxidation and increased oxidative stress, both of which can inhibit insulin signaling and exacerbate insulin resistance (Khan et al., 2019). Insulin activates the insulin receptor substrate (IRS) family of proteins by binding to its receptor. Y-Phosphorylated IRS proteins recruit and activate phosphatidylinositol 3-kinase (PI3K). PI3K converts phosphatidylinositol 4,5-bisphosphate (PIP2) to phosphatidylinositol 3,4,5-trisphosphate (PIP3), which further activates Akt (Protein Kinase B). Activated Akt regulates glucose uptake, metabolism, and storage by phosphorylating a variety of downstream target proteins. Akt facilitates the translocation of glucose transporter protein 4 (GLUT4) to the cell membrane by phosphorylating AS160 (Akt substrate 160 kDa), which increases cellular glucose uptake. Akt promotes glycogen synthesis by phosphorylating and inhibiting glycogen synthase kinase 3 (GSK-3β). Inhibition of GSK-3β allows glycogen synthase to remain active. Akt modulates lipid metabolism by regulating the activity of enzymes related to lipid synthesis and lipolysis (Huang et al., 2018).

### 3.3 Inflammatory response

In the progression of MASLD, hepatocellular lipid accumulation and the lipotoxic microenvironment drive aberrant activation of core inflammatory signaling pathways. Activation of the hepatic innate immune system (e.g., Kupffer cells) and pattern recognition receptors (e.g., TLR4 and the NLRP3 inflammasome) initiates downstream signaling cascades including c-Jun N-terminal kinase (JNK) and nuclear factor κB (NF-κB), which stimulate the secretion of pro-inflammatory cytokines (TNF-α, IL-6, IL-1β) and chemokines (Luedde and Schwabe, 2011; Gehrke and Schattenberg, 2020), thereby promoting the development and progression of MASLD. Not only does it directly drive hepatocellular damage and the progression from steatosis to MASH., but it also spreads to peripheral tissues through the circulatory system, interfering with insulin signaling, promoting vascular endothelial dysfunction and a low-grade chronic inflammatory state, which ultimately leads to metabolic syndrome-related complications such as type 2 diabetes mellitus and atherosclerosis. Additionally, hepatokines (e.g., Fetuin A, Selenoprotein P) act as molecular links between inflammation and metabolism, further aggravating insulin resistance and multiorgan metabolic dysfunction, forming a vicious cycle of “liver-peripheral organ” inflammatory cascade (Robinson et al., 2016). Therefore, targeting the hepatic inflammatory signaling regulatory network may provide a new strategy for the treatment of MASLD and its systemic complications.

### 3.4 Oxidative stress

Nuclear factor E2-related factor 2 (Nrf2) acts as the central regulator of the oxidative stress network in nonalcoholic fatty liver disease (MASLD). Activation of Nrf2 signaling critically influences disease regression by targeting multiple pathways (Ye et al., 2023). At the molecular level, Nrf2 transcriptionally activates a battery of cytoprotective genes, including Phase II detoxifying enzymes like glutathione S-transferase (GST), heme oxygenase-1 (HO-1), and key antioxidant enzymes such as glutathione peroxidase 4 (GPX4). This is achieved through the binding of Nrf2 (in complex with small Maf proteins) to the antioxidant response element (ARE) located in the promoters of these target genes. The induction of these Nrf2 target genes significantly enhances the antioxidant capacity and redox homeostasis within hepatocytes. Collectively, they mitigate oxidative damage by scavenging excess reactive oxygen species (ROS) and reducing the formation of cytotoxic lipid peroxidation end-products, such as malondialdehyde (MDA) (Xu et al., 2019). Furthermore, Nrf2 enhances mitochondrial biogenesis and function by activating pathways involved in lipid metabolism, such as the PPARγ/PGC-1α axis. This improvement in mitochondrial efficiency contributes to reduced intrahepatic lipid accumulation and improved insulin sensitivity. Consequently, this metabolic reprogramming attenuates fundamental drivers of oxidative stress. Recent studies have further revealed that the inhibitory effect of Nrf2 on ferroptosis constitutes an important addition to its hepatoprotective mechanism. Ferroptosis is a non-apoptotic cell death mode dominated by iron-dependent lipid peroxidation, and its molecular characteristics are highly correlated with the progression from MASLD to MASH. Nrf2 inhibits lipid reactive oxygen species generation and iron overload by up-regulating the expression of GPX4 and regulating iron-metabolism-related proteins (e.g., Ferritin, FPN1), thus blocking the activation of the iron death signaling pathway. This cross-pathway regulation model provides a new molecular target for precise intervention in MASLD (Bataille and Manautou, 2012).

### 3.5 Autophagy regulation

Autophagy, a conserved cellular degradation and recycling mechanism, is crucial for maintaining intracellular homeostasis and coping with metabolic stress. In the liver, autophagy regulates lipid metabolism through the selective breakdown of lipid droplets (lipophagy) as well as through modulation of fatty acid β-oxidation and lipid droplet formation (Singh et al., 2009).

In hepatocytes, autophagy exerts protective functions via at least two pathways. First, it degrades aggregates of misfolded proteins, thereby reducing proteotoxic stress and preserving intracellular proteostasis. Second, it mediates mitophagy—the selective clearance of damaged mitochondria—to prevent excessive reactive oxygen species (ROS) production, thereby helping to mitigate oxidative stress and dampen inflammatory response (Filali-Mouncef et al., 2022). Autophagy activation may supply energy to hepatic stellate cells (HSCs), promote their activation, and result in fibrogenesis. Moreover, if autophagy is activated when MASLD has advanced to MASH or advanced liver cancer, it may not yield significant therapeutic effects and could even facilitate the survival of tumor cells. The core molecular machinery of autophagy, orchestrated by autophagy-related (ATG) genes, responds to cellular stress through a complex signaling network (Qian et al., 2021). The formation of autophagosomes requires two ubiquitin-like conjugation cascades: the ATG12-ATG5 cascade and the LC3 cascade. LC3-II is a marker of autophagosomes and is involved in autophagosome formation and cargo recognition (Martinez-Lopez and Singh, 2015). For example, deletion of ATG7 or ATG5 in hepatocytes inhibits lipophagy and exacerbates hepatic steatosis, whereas activation of autophagy in hepatic stellate cells (HSCs) promotes their activation through the release of fatty acids, driving collagen deposition and fibrosis. The transcription factors TFEB and TFE3 are potential therapeutic targets by regulating autophagy-related genes (e.g., ATG7, LC3) and lipid metabolic pathways. In addition, ubiquitination modification of lipid droplet surface proteins (e.g., AUP1, SPART) and involvement of Rab GTPases (e.g., RAB7, RAB10) further revealed the molecular regulatory network of lipophagy (Ueno and Komatsu, 2017; Filali-Mouncef et al., 2022). Therefore, precise and context-dependent modulation of autophagy represents a critical therapeutic strategy for MASLD.

### 3.6 Gut microbiota and associated metabolites

Accumulating evidence underscores the critical role of the gut microbiota in the development and progression of MASLD. Its impact is mediated primarily through the gut-liver axis, where it modulates host metabolic, immune, and inflammatory responses, thereby driving MASLD pathogenesis. Studies have shown that the composition and function of the gut microbiota are significantly altered in patients with MASLD, as evidenced by dysbiosis of the gut microbiota, including a decrease in beneficial bacteria (e.g., *Akkermansia muciniphila*) and an increase in harmful bacteria (e.g., *Escherichia coli*). This dysbiosis leads to impaired intestinal barrier function and increased intestinal permeability, allowing bacteria and their metabolites (e.g., lipopolysaccharide [LPS]) to enter the liver, triggering an inflammatory response and hepatocyte damage (Song and Zhang, 2022). Metabolites of the gut microbiota, such as short-chain fatty acids (SCFAs), bile acids (BAs), and microbe-dependent metabolites like trimethylamine N-oxide (TMAO), also play an important role in the development of MASLD. For example, SCFAs regulate hepatic lipid metabolism and inflammatory responses through activation of G protein-coupled receptors (e.g., GPR43 and GPR41), whereas bile acids regulate hepatic metabolic functions by affecting the signaling pathways of farnesoid X receptor (FXR) and Takeda G protein-coupled receptor 5 (TGR5) (Canfora et al., 2019). In addition, endotoxins (e.g., LPS) produced by the gut microbiota can induce hepatic inflammatory responses by activating Toll-like receptors (TLRs) signaling pathways (Han et al., 2023). Modulation of the gut microbiota has emerged as a potential strategy for MASLD treatment.

**FIGURE 3:**
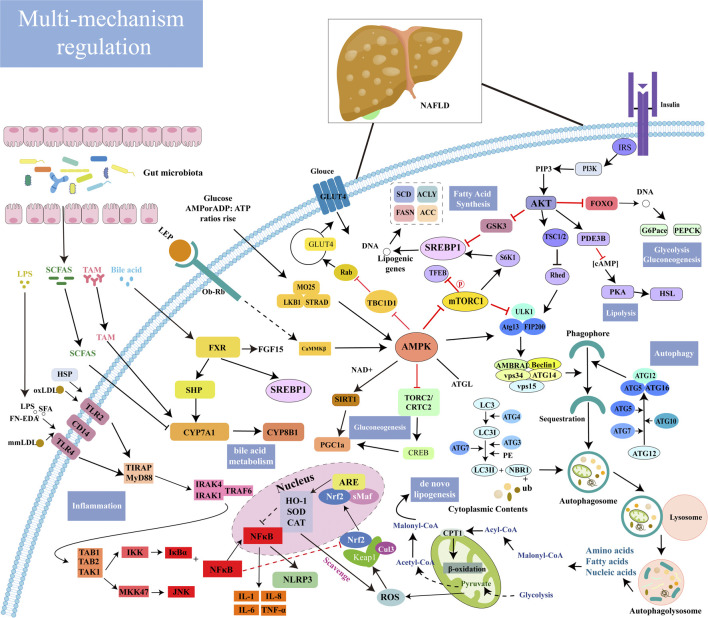
Mechanisms of Metabolic dysfunction-associated steatotic liver disease (MASLD) diagram.

The pathological progression of MASLD is governed by a complex regulatory network characterized by the interweaving of multiple pathways and the coupling of several functional modules. Its core feature lies in the fact that six major modules—metabolic homeostasis, lipid synthesis and catabolism, inflammatory cascade, oxidative stress defense, autophagy, and gut–liver axis regulation—do not operate independently. Instead, they engage in extensive cross‐talk and form positive and negative feedback loops through key nodes such as AMPK, AKT, and mTORC1, constituting a highly integrated molecular regulatory system. This network‐based pathological mechanism suggests that interventions for MASLD require a multi‐target, systemic integrative strategy rather than linear regulation of a single pathway (Figure 3).

## 4 Therapeutic mechanisms of flavonoids

### 4.1 Energy metabolism and insulin resistance

#### 4.1.1 Vitexin

Vitexin is widely present in numerous edible and medicinal plants, including *Crataegus rhipidophylla* Gand., *Vigna radiata* (L.) R. Wilczek, and *Trigonella foenum-graecum* L. This compound exhibits multiple biological activities, including antioxidant, anti-inflammatory, anti-cancer, lipid-lowering, glucose metabolism-regulating, and hepatoprotective effects (Peng et al., 2021). Vitexin binds to the extracellular cytokine receptor homology (CRH) domain of leptin receptor (LepR), suppressing lipogenesis primarily through downregulation of SREBP-1c and its downstream targets (e.g., FAS, ACC). Simultaneously, it activates AMPK signaling to enhance fatty acid oxidation, while concurrently improving hepatic insulin sensitivity via IRS-1/AKT pathway activation. These dual mechanisms synergistically modulate lipid and glucose metabolism in high-fat diet (HFD)-fed mice (Inamdar et al., 2019).

#### 4.1.2 Calycosin

Calycosin, derived from the root of *Astragalus membranaceus Fisch*. Ex Bunge, possesses dual anti-inflammatory and antioxidant properties (Ma et al., 2024). Calycosin specifically activates the farnesoid X receptor (FXR), thereby inhibiting the expression of key gluconeogenic enzymes (PEPK, G-6-pase) and lipid synthesis regulators (SREBP-1c, FASN) in mice fed a HFD. This reduces hepatic glucose production and triglyceride synthesis, mitigating liver injury and steatosis. Concurrently, by enhancing insulin signalling (elevating p-IRS-1/2 expression), promoting glucose transport (upregulating Glut-4), and stimulating glycogen synthesis (increasing Gsk3β), it ameliorates insulin resistance. Ultimately, this multi-faceted approach improves the pathological state of MASLD (Duan et al., 2018).

#### 4.1.3 Licochalcone A

Licochalcone A is a bioactive flavonoid compound isolated from liquorice (Glycyrrhiza uralensis Fisch. ex DC.) and widely employed in clinical practice within traditional Chinese medicine (Li M.-T. et al., 2022). Licochalcone A effectively restores hepatic lipid metabolic homeostasis, as jointly validated in mice fed a HFD and in oleic acid (OA) -induced HepG2 cells. It not only alleviates MASLD by activating the Sirt1/AMPK core pathway but also reduces fatty acid and triglyceride production by inhibiting lipid synthesis transcription factors such as SREBP-1c and PPAR-γ, along with FAS expression. It promotes ATGL and pHSL expression and accelerates lipid breakdown via CPT-1-mediated fatty acid β-oxidation. It blocks hepatic fatty acid uptake to reduce lipid accumulation; subsequently, it simultaneously regulates leptin and adiponectin balance to lower fasting blood glucose and insulin levels, thereby improving insulin resistance. Ultimately mitigating hepatic steatosis and liver injury (Liou et al., 2019).

#### 4.1.4 Chrysin

Chrysin is a naturally occurring compound extracted from various plants, including *Passiflora caerulea* L.*, Passiflora incarnata* L, *Scutellaria immaculata* Nevski ex Ju (Kurkiewicz et al., 2025). Mechanistic studies reveal that. chrysin activates AMPK/LKB1 signaling, thereby inhibiting mTOR/SREBP-1c-mediated hepatic lipogenesis. Concurrently, it promotes mitochondrial biogenesis by upregulating key regulators PGC-1α, NRF1, and Tfam, while enhancing cellular antioxidant defenses. These integrated actions collectively ameliorate obesity-related metabolic dysregulation in HFD-induced models, as evidenced by significant improvements in multiple physiological parameters (Oriquat et al., 2023).

#### 4.1.5 Baicalin

Baicalin is one of the most abundant flavonoid compounds in the dried roots of *Scutellaria baicalensis* Georgi, a plant belonging to the genus Scutellaria. Research has confirmed that baicalin possesses multiple biological activities, including anti-inflammatory, antitumour, antioxidant and hepatoprotective (Wen et al., 2023). Baicalin significantly improves hepatic steatosis, oxidative stress, and inflammatory response in HFD-fed mice models by activating the AMPK signaling pathway to inhibit SREBP1-driven lipid synthesis, enhance Nrf2 antioxidant defense, and suppress the NF-κB inflammatory pathway, moreover, the AMPK inhibitor Compound C can completely reverse the aforementioned effects (Gao et al., 2023).

#### 4.1.6 Wogonin

Wogonin, a major bioactive flavonoid derived from the roots of Scutellaria baicalensis (Chinese herbal medicine Huang-Qin), has demonstrated significant therapeutic potential in liver-related diseases such as MASLD. Chen et al. (2017) demonstrated in HFD-induced MASLD mice and palmitate-stressed NCTC 1469 hepatocytes that Wogonin significantly improved disease-related indicators. Mechanistically, wogonin restored the lipotoxicity-induced decline of PPARα and its downstream target AdipoR2. PPARα knockdown by siRNA completely abolished these protective effects, including AdipoR2 upregulation, confirming that wogonin mitigates NAFLD through a PPARα/AdipoR2-dependent pathway (Chen et al., 2017)

#### 4.1.7 Naringin

Naringin, a flavone glycoside predominantly found in. Sarkar et al. (2025) showed in HFD-induced NAFLD rats that naringin improved metabolic parameters, hepatic steatosis, and fibrosis by activating AMPK and inhibiting SREBP-1C, thereby suppressing de novo lipogenesis. Additionally, naringin restored antioxidant enzyme activities and alleviated ER stress and mitochondrial damage, highlighting a multi-target protective mechanism against NAFLD (Sarkar et al., 2025)

### 4.2 Oxidative stress and immune regulation

#### 4.2.1 Hesperidin

Hesperidin is a *Citrus* L. flavonoid exhibiting multiple pharmacological activities, including antitumour, anti-inflammatory, antioxidant (Wang et al., 2024). Hesperetin enhances cellular antioxidant capacity in association with activation of the PI3K/AKT-Nrf2 pathway and inhibits the activation of the NF-κB inflammatory pathway. This facilitates multi-target intervention in oxidative stress and metabolic disorders in oleic acid-induced HepG2 cells and HFD-fed Wistar rats, providing a basis for cross-pathway synergy in the pathological regulation of MASLD (Li et al., 2021).

#### 4.2.2 Fisetin

Fisetin is commonly found in *Fragaria* L., *Allium* L. and Cucurbitaceae Juss., as well as in various trees and shrubs belonging to the Fabaceae and Anacardiaceae families (Kashyap et al., 2019). Fisetin alleviates oxidative stress and mitochondrial dysfunction in palmitic acid (PA)-treated human hepatocyte line L02 and mouse hepatocytes AML12 by inhibiting GRP78, a key factor in endoplasmic reticulum (ER) stress, and activating the AMPK-Nrf2 pathway. This mechanism operates by downregulating the expression of key ER stress molecules such as GRP78 and CHOP, thereby blocking the oxidative stress and cellular damage they induce. Concurrently, it activates the Nrf2 antioxidant pathway, reducing cellular and mitochondrial reactive oxygen species (ROS) production while enhancing antioxidant capacity via SOD and GSH. This approach also improves mitochondrial membrane potential and ATP synthesis, thereby reversing mitochondrial dysfunction. Furthermore, through crosstalk between the endoplasmic reticulum stress and oxidative stress pathways, it further inhibits hepatocyte apoptosis, the release of inflammatory mediators such as TNF-α and IL-1β, and the expression of lipid synthesis genes such as FASN and SCD1. This ultimately alleviates lipid deposition, inflammatory infiltration, and cellular damage in liver tissue, thereby achieving the prevention and treatment of MASLD (Dai et al., 2022).

#### 4.2.3 Trilobatin

Trilobatin is a glycosylated dihydrochalcone isolated from the leaves of the Chinese sweet tea *Lithocarpus polystachyus* (Wall. ex A. DC.) Rehder. Trilobatin inhibits α-glucosidase and α-amylase activity associated with type 2 diabetes and also possesses antioxidant and anti-inflammatory properties (Fan et al., 2015). Trilobatin effectively suppressed the cascading amplification of metabolic inflammation in mice fed a HFD and injected with streptozotocin (STZ). This was achieved by inhibiting key metabolites of NLRP3 and its upstream p65 NF-κB protein expression, suppressing caspase-1 activation and GSDMD N-terminal cleavage, thereby reducing the release of IL-1β and IL-18. This approach suppresses hepatic inflammatory responses and pyroptosis while concurrently improving glucose metabolism disorders, mitigating lipid deposition and hepatic fibrosis, ultimately achieving dual protection of liver function (Zhang et al., 2022).

#### 4.2.4 Naringenin

Naringenin is a predominant flavonoid compound found in natural plants, abundant in citrus fruits, particularly *Citrus* L. This compound exhibits extensive biological and pharmacological activity, with mounting evidence indicating its significant efficacy in controlling various inflammation-related diseases such as acute hepatitis, fibrosis, and cancer (Zeng et al., 2018; Wang et al., 2020). Naringenin treats MASLD through anti-inflammatory mechanisms, as demonstrated in studies by Hua et al. and Wang et al. by inhibiting the NF-κB pathway and suppressing the release of pro-inflammatory factors such as TNF-α and IL-6. The distinction lies in the fact that Hua et al. employed an ApoE^−/−^ aged metabolic syndrome mouse model, where the anti-inflammatory effect depended on NF-κB deacetylation mediated by SIRT1 activation, concurrently improving lipid metabolism, fibrosis, and hepatic cellular senescence. Wang employed multi-model validation—including an *in vivo* MCD diet-induced wild-type mouse model and *in vitro* HepG2 and Kupffer cell models stimulated with LPS combined with oleic acid—to clarify the anti-inflammatory mechanism. This mechanism involves direct downregulation of the NLRP3 inflammasome, blocking IL-1β/IL-18 maturation, and is NLRP3-dependent. It exhibited no significant hypolipidemic or anti-inflammatory effects in NLRP3 knockout mice (NLRP3^−/−^). However, upon adenoviral supplementation of NLRP3 protein in NLRP3^−/−^ hepatocytes, the anti-lipid deposition effects of naringenin were restored (Wang et al., 2020; Hua et al., 2021).

#### 4.2.5 Sappanone A

Sappanone A is a homoisoflavanone compound that is isolated from the heartwood of *Caesalpinia sappan* L., it possesses antioxidant and anti-inflammatory properties (Lee et al., 2015). Sappanone A significantly promotes hepatic lipid efflux and inhibits lipotoxicity in MCD diet-induced MASH (pre- MASH) mice model by upregulating the expression of the lipid transporter Mup3. Mup3, functioning as an apolipoprotein, facilitates lipid transport and degradation, reducing hepatic triglyceride and total cholesterol accumulation alongside palmitate-induced lipotoxicity. Concurrently, it inhibits M1 macrophage infiltration and the release of pro-inflammatory mediators such as TNFα and IL-1β, diminishes collagen deposition in hepatic tissue (downregulating Col1, α-SMA and other genes), ultimately blocking the “lipotoxicity-inflammation-fibrosis” progression of MASH through multiple pathways. Its high-dose efficacy is comparable to silymarin (Zhu et al., 2025).

#### 4.2.6 Breviscapine

Breviscapine is a mixture of flavonoid glycosides isolated from Chinese botanical drugs, including *Erigeron breviscapus* (Vaniot) Hand.-Mazz. Breviscapine has been reported to have various biological activities, including anti-oxidant, anticancer, anti-degenerative and anti-angiogenesis effects (Liu et al., 2018). Breviscapine exerts its anti- MASH effects in HFHC diet-fed mice models by directly binding to and inhibiting the phosphorylation of TGF-β-activated kinase 1 (TAK1). On the one hand, it blocks downstream MAPK (JNK/p38) and NF-κB signalling pathways, reducing the release of pro-inflammatory factors (TNFα) and inflammatory cell infiltration, thereby alleviating hepatic inflammation. Simultaneously, It downregulates fatty acid synthesis/uptake-related genes (FASN, CD36, etc.) and upregulating fatty acid β-oxidation genes (PPARα, Cpt1α, etc.) to improve lipid metabolism disorders and reduce hepatic TG and TC accumulation. It also inhibits the expression of pro-fibrotic genes (Col1a1, Ctgf, etc.), diminishing collagen deposition, thereby multidimensionally suppressing MASH progression (Lan et al., 2022).

#### 4.2.7 Eriocitrin (ER)

ER exerts anti-fibrotic effects in a thioacetamide (TAA)-induced mouse model of hepatic fibrosis and in transforming growth factor-β (TGF-β)-activated hepatic stellate cells (HSCs) by targeting the peroxisome PPARα-mediated inflammatory regulatory pathway. On one hand, ER upregulates PPARα transcription and protein expression, directly inhibiting NLRP1/NLRC4 inflammasome activation. This reduces the maturation and release of pro-inflammatory cytokines such as IL-1β and IL-6, whilst simultaneously downregulating the expression of inflammation-associated molecules including IL1R1 and IL-23, thereby blocking the inflammatory cascade. It also inhibits lipopolysaccharide/adenosine triphosphate (LPS/ATP)-induced activation of bone marrow-derived macrophages (BMDMs), diminishes macrophage-derived proinflammatory signalling, and reduces neutrophil infiltration alongside the expression of neutrophil extracellular trap (NET) markers, thereby preventing inflammatory escalation. Conversely, ER downregulates the expression of fibrotic genes such as Col1a1 and Acta2/α-SMA, as well as other fibrosis-related genes, inhibiting HSC activation and abnormal deposition of extracellular matrix (ECM), and improving collagen accumulation in liver tissue.

#### 4.2.8 Acacetin

Acacetin blocks ferroptosis induced by oleic acid (OA) and lipopolysaccharide (LPS) in HepG2 cells by upregulating the ferroptosis inhibitory gene GPX4 and downregulating the expression of the pro-ferroptosis genes ACSL4 and FTH1. It also significantly inhibits the protein levels of the key endoplasmic reticulum stress (ER stress) factors ATF6 and CHOP, achieving a synergistic intervention in oxidative damage and lipotoxicity in C57BL/6J mice fed a HFD and in cell models (Jiang et al., 2023).

### 4.3 Regulation of autophagy

#### 4.3.1 Buddleoside

Buddleoside (Bud), a natural flavonoid compound, has been isolated from various plants mainly belonging to the Asteraceae and Lamiaceae, Scrophulariaceae and *Valerianaceae* families. Bud significantly alleviates MASH lesions in mice induced by HFHC or HFD diets. The specific mechanism involves Bud activating AMPK and inhibiting mTORC1 both *in vivo* and *in vitro*, thereby enhancing TFEB transcriptional activity and autophagy flux. This mechanism is completely abolished upon inhibition of AMPK or liver-specific knockout of TFEB. Molecular docking and mutation experiments further confirm that Bud directly binds to the AMPKβ1 subunit (PRKAB1) via residues Val81, Arg83, and Ser108. This activation of AMPK subsequently promotes RPTOR phosphorylation, thereby inhibiting MTORC1 activity. Ultimately, this leads to an improvement in the MASH pathological phenotype via the TFEB-mediated autophagy-lysosomal pathway (Chen et al., 2025).

#### 4.3.2 Galangin

Galangin is an important flavonoid with natural bioactivity, abundant in *Alpinia zerumbet* (Pers.) B.L.Burtt & R.M.Sm. and propolis. To date, multiple biological activities of galangin have been identified, including anti-inflammatory, antibacterial, antioxidant stress and anti-ageing, anti-fibrotic, and anti-hypertensive effects (Wang D. et al., 2023). In a combined study of C57BL/6J mice on a HFD and FFA-induced HepG2 cells, galangin activated the AMPK signalling pathway, upregulating autophagy-related proteins such as Beclin1, Atg3, LC3-II/LC3-I, while simultaneously inhibiting the autophagy-negative regulator mTOR, thereby promoting autophagy. It downregulates CD36, SREBP1c, and ChREBP to suppress lipid uptake and synthesis, whilst upregulating PPARα and CPT1a to enhance fatty acid oxidation. This ultimately achieves dynamic equilibrium in lipid metabolism, thereby alleviating hepatic steatosis and liver injury. This effect is blocked by the autophagy inhibitor 3-MA, further confirming that galangin-induced autophagy activation constitutes the core mechanism through which it regulates lipid metabolism and ameliorates MASLD (Zhang et al., 2020).

#### 4.3.3 Didymin

Didymin is a flavonoid compound isolated and identified from *Origanum vulgare* L. (Wei et al., 2017). Didymin exerts its anti-MASLD effects by dual activation of Sirt1: on the one hand, it directly binds to Sirt1 and enhances its deacetylase activity; on the other hand, it upregulates Sirt1 expression by activating the FoxO3a transcription factor. The activated Sirt1 subsequently improves MASLD pathology through three major pathways: deacetylating PGC-1α to promote NRF1/TFAM expression, thereby enhancing mitochondrial biogenesis and respiratory function; deacetylating FoxO3a to regulate genes such as Atg5/Beclin1, activating lipophagy and reducing hepatic triglyceride accumulation; while simultaneously enhancing lipophagy to clear damaged substances and inhibiting Bax/caspase3-mediated hepatocyte apoptosis. These effects were validated both in AML12 cells treated with palmitate (*in vitro*) and in MASLD mice induced by a HFD diet (*in vivo*). Furthermore, Sirt1 inhibitors (such as EX-527) completely reversed this therapeutic effect, confirming Sirt1 as the core target through which Didymin ameliorates MASLD (Yang et al., 2023).

#### 4.3.4 Formononetin

Formononetin, a phytoestrogen derived from the traditional Chinese medicinal botanical drug *Trifolium pratense* L., possesses anti-tumor, hepatoprotective, and neuroprotective activities (Yu et al., 2022). Formononetin, in combined treatment studies involving C57BL/6J mice fed a HFD and HepG2 cells treated with FFA, promotes TFEB nuclear translocation by activating AMPK and relieving mTORC1 inhibition of transcription factor EB (TFEB). Intranuclear TFEB further upregulates expression of lysosomal biogenesis-related genes (LAMP1, ATP6V1A) and the mitochondrial function regulator PGC1α, increasing functional lysosome numbers, repairing blocked autophagic flux (alleviating LC3B-II accumulation and p62 clearance defects), and enhancing autophagosome-lysosome fusion efficiency; Concurrently, it activates lipophagy, promoting the degradation of lipid droplets into free fatty acids. Subsequently, by upregulating PPARα and CPT1α, it accelerates fatty acid β-oxidation, ultimately reducing hepatic lipid accumulation while simultaneously improving serum dyslipidaemia, insulin resistance, and hepatic dysfunction. Knockdown experiments confirmed TFEB as the key target mediating Formononetin effects, providing theoretical support for natural drug interventions in MASLD (Wang et al., 2019).

#### 4.3.5 Puerarin

Puerarin is the major bioactive metabolite isolated from the root of the *Pueraria lobata* (Willd.) Ohwi, it has been extensively applied in the treatment of cardiovascular and cerebrovascular diseases, diabetes and its complications, osteonecrosis, Parkinson’s disease, Alzheimer’s disease, endometriosis, and cancer (Zhou et al., 2014). Puerarin initiates macrophage autophagy by activating the AMPK-mTOR-ULK1 signalling pathway, thereby downregulating plasminogen activator inhibitor-1 (PAI-1), activates autophagy, and modulates the Stat3/Hif-1α/PI3K/AKT signalling network. This synergistically remodels the M1/M2 balance in macrophages within HepG2 cells treated with free fatty acids and in C57BL/6 mice subjected to AMLN diet-induced inflammation (Fang et al., 2024).

### 4.4 Gut microbiota and associated metabolites

#### 4.4.1 Rutin

Rutin ameliorated lipid metabolism disorders in high-glucose-fed db/db mice and palmitic acid (PA)-treated HepG2 cells through activation of the AMPK/SREBP1 pathway, and significantly modulated the composition of the intestinal flora. Rutin reduced gut microbiota dysregulation, such as the Firmicutes/Bacteroidetes ratio, and synergistically inhibited hepatic lipid accumulation through gut flora (Liu et al., 2024).

#### 4.4.2 Myricetin

Myricetin (Myr) is a flavanone compound originally isolated from the bark of myricetin. Myricetin plays a significant role in metabolic disorders due to its anti-inflammatory, antioxidant, and lipid-lowering properties. Myricetin, which activates AMPK phosphorylation and inhibits ACC/HMGCR-mediated lipid synthesis by increasing the abundance of butyric acid-producing bacteria (e.g., Lachnospiraceae, *Allobaculum*), significantly reduces plasma LPS levels in a HFD-induced rat model of MASLD, and inhibits TLR4/NF-κB inflammatory pathway and enhance intestinal barrier function (ZO-1) to ameliorate hepatic lipotoxicity at the level of colony metabolites (Sun W.-L. et al., 2021).

#### 4.4.3 Hesperidin

Hesperidin was first isolated from citrus peel by the French chemist Lebreton. This compound has also been confirmed to exist in plants of the Rutaceae Juss. family, as well as in the fruit of *Monarda fistulosa* L., Musaceae Juss., *Citrus limon* (L.) Burm. f. Research has demonstrated its therapeutic effects on obesity-related conditions (Xiong et al., 2019). Hesperidin improves HFD-fed C57BL/6J mice through multidimensional synergistic effects, specifically through the following mechanisms: Firstly, Hesperidin selectively remodels the gut microbiota, increasing the abundance of beneficial bacteria such as Bacteroidetes, Verrucomicrobium, and Oscillospirales while reducing the Firmicutes/Bacteroidetes ratio. These microbial groups enhance gut-liver axis function by producing short-chain fatty acids (SCFAs) or regulating metabolites like sphingosine. In addition, hesperidin regulates hepatic metabolism and gene expression by enhancing arginine biosynthesis (increasing metabolites such as DL-ornithine and spermidine) and mitochondrial oxidation (upregulating acetyl-CoA, 3-methyl-2 -oxo-pentanoic acid (MOVA) to alleviate inflammation and promote energy metabolism. It also reduces hepatic lipid accumulation by activating PPARα and its downstream fatty acid oxidation genes (CPT1α) while inhibiting lipogenic genes (SREBP-1C). Furthermore, the beneficial bacteria enriched by hesperidin positively correlate with hepatoprotective metabolites, collectively forming a “gut microbiota - liver metabolism” regulatory cycle, thereby achieving therapeutic effects for MASLD (Li X. et al., 2022).

### 4.5 Multiple mechanisms to regulate

#### 4.5.1 Genistein

Genistein, an isoflavone derived from *Glycine* max (L.) Merr, exerts therapeutic effects on MASLD through multiple synergistic pathways. In terms of metabolic regulation, Liu et al. (2017) observed in a HFS(high-fat sucrose) diet rat model that genistein activates the AMPK pathway, thereby upregulating p-AMPK and p-ACC protein levels while downregulating mRNA expression of SREBP-1 and lipid synthesis genes FAS and GPAT. Concurrently, it enhances PPARα, CPT-1, and ACO-mediated mitochondrial fatty acid β-oxidation, thereby achieving effective regulation of lipid metabolism; In terms of anti-inflammatory and antioxidant effects, Yin et al. (2019)(Yin et al., 2019) demonstrated in a MASH rat model that it downregulates TLR4 protein and mRNA expression, reduces endotoxin and hepatic tissue TNF-α levels, alleviates inflammatory infiltration, and lowers the MASLD activity score (NAS); Wang et al. (2018) demonstrated in HFD-induced mice and HepG2 cells that it directly targets COX-1 activity, inhibits thromboxane A2 (TXA2) biosynthesis and TBXA2R expression, blocks TXA2-mediated insulin signalling inhibition, enhances IR and IRS1 phosphorylation levels, and improves insulin resistance to delay MASLD progression. Amanat et al. (2018a) further validated in a clinical trial involving MASLD patients that it reduces serum insulin levels and the HOMA-IR index, decreases triglycerides and body fat percentage, and lowers serum levels of inflammatory factors such as TNF-α and IL-6, as well as malondialdehyde (MDA). These studies, spanning from *in vitro* cellular models and animal models to the clinical level, comprehensively reveal that genistein exerts protective and therapeutic effects on MASLD through multi-dimensional mechanisms. These include regulating metabolic pathways, inhibiting inflammatory responses, alleviating oxidative stress, and targeting the thromboxane A2 (TXA2) pathway.

#### 4.5.2 Kaempferol

Kaempferol is a major flavonoid compound widely present in fruits, vegetables and traditional Chinese medicinal botanical drugs. Research has demonstrated that kaempferol treats MASLD through multi-pathway, multi-target synergistic action. (Yao et al., 2024). Kaempferol in the MASH mouse model established by Lu et al. (2022), kaempferol exerts its effects “partly through regulating bile acid metabolism and associated gene expression. On the one hand, it elevates serum β-MCA, UDCA, and ωMCA, and hepatic 12-DHCA levels, while reducing serum secondary bile acids such as TDCA, THDCA, and TUDCA. This corrects abnormalities in indicators including total CA% and the primary BAs/secondary BAs ratio in the liver, thereby maintaining bile acid pool homeostasis. Kaempferol can also promotes CYP27A1, NTCP mRNA expression, reversing HFD-induced elevation of CYP7A1 and reduction of CYP8B1 and MRP3, thereby repairing bile acid synthesis and transport pathways. Liu et al. (2021) conducted a combined analysis of network pharmacology, in HepG2 cells (*in vitro*) and high-fat diet-induced rats (*in vivo*), demonstrating that kaempferol inhibits the nuclear transcription activity of the NF-κB pathway. This occurs by reducing IκB degradation, suppressing NF-κB phosphorylation and nuclear translocation, thereby decreasing the expression of inflammatory mediators (TNFα, IL6) and blocking inflammatory responses to prevent the progression from simple fatty liver to non-alcoholic steatohepatitis. Concurrently, by targeting insulin resistance through pathways such as AKT1 and INS, and mitigating oxidative stress via pathways including CAT and HIF1A, it synergistically reduces hepatic lipid accumulation, improves hepatic steatosis and fibrosis, thereby achieving multi-target prevention and treatment of MASLD.

#### 4.5.3 Dihydromyricetin

Dihydromyricetin (DHM), is abundant in Ampelopsis grossedentata (Hand.-Mazz.) W.T. Wang (Vitaceae) (20%–30%, w/w), Its tender stems and leaves are widely used as vine tea in southern China (Chen et al., 2021). It demonstrates a multi-pathway synergistic mechanism in the treatment of MASLD, with its efficacy validated across multiple models by various research teams. In HFD-induced MASLD rats and palmitic acid (PA)-treated HepG2 cell models, DHM activates hepatic autophagy via the AMPK/PGC-1α/PPARα pathway (upregulating Beclin1, ATG5, and LC3-II) to promote lipid degradation. Concurrently, it modulates key molecules in glucose metabolism (increasing GLUT2 expression while decreasing G6Pase and PEPCK expression) to improve insulin resistance. AMPK/PGC-1α and PPARα knockdown reduced the impact of DHM on hepatic autophagy, IR and accumulation of hepatic lipid, demonstrated its therapeutic efficacy for MASLD (Yang et al., 2024). In CCl4-induced liver fibrosis in C57BL/6 mice and TGF-β1-activated LX2 hepatic stellate cells (HSCs), DHM directly inhibits HSC activation by inducing HSC autophagy (upregulating LC3B-II and Beclin1, reducing SQSTM1, with the autophagy inhibitor 3-MA blocking this effect), and secondly by promoting IFN-γ secretion from NK cells via the AhR-NF-κB/STAT3 pathway, thereby enhancing their cytotoxic activity against HSCs. Concurrently, it reduces the expression of fibrosis markers such as Col-1α1, TIMP-1, and α-SMA, mitigating collagen deposition (Zhou et al., 2021). Similarly, in a CCl_4_-induced liver fibrosis model in C57BL/6 mice, DHM further remodels the gut microbiota composition (reducing pro-inflammatory groups such as Bacilli, Gemmatimonadete, and Lactobacillaceae while increasing beneficial groups like Pseudomonadales and Planococcaceae), modulates hepatic biotin metabolism pathways (by lowering adipate levels), while simultaneously suppressing mRNA expression of inflammatory and fibrotic markers including IL-2, IL-6, TGF-β, and Col-I. This approach reduces α-SMA-positive areas, thereby further refining the therapeutic mechanism for liver fibrosis (Gao et al., 2025). In HFD-induced MASLD mice, SIRT3 knockout (SIRT3KO) mice, and in a PA-treated hepatocyte model, DHM enhances SIRT3 expression via the AMPK-PGC1α/ERRα pathway. This improves mitochondrial respiratory chain function (upregulating MT-CO1/MT-ATP6), activates SOD2 antioxidant activity to reduce mitochondrial reactive oxygen species (mtROS), and disrupts the “lipid accumulation - mitochondrial dysfunction’ cycle. Furthermore, SIRT3 knockdown completely abrogated the protective effects of DHM (Zeng et al., 2019).

#### 4.5.4 Quercetin

Quercetin promotes autophagosome formation in FFA (oleic acid/palmitic acid)-induced HepG2 cells and HFD-fed mice through activation of the AMPK signaling pathway and specifically activates mitochondrial autophagy (PINK1/Parkin pathway), effectively removing dysfunctional mitochondria and simultaneously ameliorating oxidative stress and lipid metabolism disorders (Cao P. et al., 2023). Quercetin inhibits the TLR4-NF-κB pathway and NLRP3 inflammatory vesicle activity (Caspase-1), synchronously relieves endoplasmic reticulum stress (GRP78, CHOP) and enhances intestinal tract in high-fat dietary mice by lowering the phylum Thick-walled Bacteria/phytobacteriophage (F/B) ratio and reducing the abundance of pathogenic bacteria such as *Helicobacter*. The simultaneous alleviation of endoplasmic reticulum stress (GRP78, CHOP) and enhancement of intestinal barrier integrity (occludin, intestinal alkaline phosphatase) revealed the central role of colony remodeling in interfering with the pathological process of MASLD through the multi-targets of the “gut-hepatic axis” (Porras et al., 2017).

#### 4.5.5 Alpinetin

Alpinetin is a plant flavonoid isolated from Alpinia katsumadai Hayata with antioxidant and anti-inflammatory properties (Dong et al., 2022). Alpinetin activates the Nrf2 pathway and upregulates its downstream antioxidant enzymes (SOD1, HO-1), thereby inhibiting the TLR4/NF-κB inflammatory signalling pathway to reduce secretion of pro-inflammatory cytokines such as IL-1β and TNF-α, thereby alleviating inflammatory responses; It downregulates lipid synthesis-related molecules including SCD1, FAS, and SREBP-1c, whilst upregulating PPARα to improve lipid metabolism disorders. Concurrently, it inhibits JNK phosphorylation and restores IRS1 phosphorylation levels, thereby alleviating insulin resistance. This ameliorates oxidative stress and lipid metabolism disorders in C57BL/6 mice fed a high-fat diet (HFD) and in HL-7702 hepatocytes induced by high-concentration fructose (Zhou et al., 2018).

#### 4.5.6 Hyperoside

Hyperoside, as a natural flavanol glycoside compound, exerts therapeutic effects through multi-targeted and multi-pathway synergistic mechanisms in a high-fat diet-induced MASLD model, Wang et al. (2025) discovered in a HFD-induced rat model that it directly binds to and activates FXR, thereby inhibiting SCD1 and SREBP1 to suppress *de novo* lipogenesis, promote fatty acid oxidation, and suppress intestinal BSH (bile-salt hydrolase)-producing microbes to elevate conjugated bile acid levels and alleviate hepatotoxicity. While independently inhibiting ACLY activity to block fatty acid and cholesterol synthesis, thereby suppressing *de novo* fatty acid synthesis; Sun B. et al. (2021) demonstrated in a combined HFD-fed mouse and Nr4A1 (nuclear receptor subfamily 4A1) knockout mouse model that hyperoside exerts a protective effect against HFD-induced MASLD. This protection occurs via Nr4A1-dependent regulation of macrophage polarisation by upregulating the expression of Nr4A1. Consequently, it reduces inflammatory infiltration in both hepatic and white adipose tissues, improves insulin resistance and alleviates hepatic fibrosis. However, in Nr4A1 knockout mice, hyperoside failed to exert protective effects or modulate macrophage polarisation.

#### 4.5.7 Bavachin

Bavachin significantly reduces lipid accumulation in C57BL/6J mice subjected to a high-fat diet (HFD) and in Huh7 cells treated with palmitic acid/oleic acid (PA/OA) by inhibiting the expression of key genes involved in fat synthesis, such as FAS, ACC, and SCD-1. Additionally, it enhances insulin signaling pathways through the phosphorylation of AKT and GSK-3β and promotes energy expenditure by activating thermogenesis-related genes (UCP1, PGC-1α), thereby achieving dual regulation of lipid and glucose metabolism (Wei et al., 2023).

#### 4.5.8 Butein

Butein intervenes in the inflammatory process through a dual regulatory mechanism: activation of the ERK/Nrf2 pathway to enhance antioxidant defenses on the one hand, and direct inhibition of the NF-κB signaling pathway on the other hand, to alleviate the simultaneous oxidative stress and inflammatory damage in the Wistar rat model of MCD diet (Alshammari et al., 2018).

#### 4.5.9 Taxifolin

In HFD-induced C57BL/6J mouse and Mc4r-deficient mouse models, Taxifolin effectively intervenes in the MASLD- MASH-Carcinoma of liver progression through a dual mechanism: on the one hand, it inhibits the SREBP1c/FAS/ACC lipid synthesis pathway in liver cells, reduces lipid deposition, downregulates inflammatory factors (TNF-α, IL-1β), and alleviates oxidative stress. On the other hand, it activates thermogenic genes (UCP1, PGC-1α) in brown adipose tissue and increases energy expenditure through FGF21 signaling. In both preventive and therapeutic regimens, Taxifolin can significantly improve Liver fatty metamorphosis, Fibrosis and Inflammatory reaction, effectively prevent the progression of MASH to Carcinoma of liver, and reduce the incidence of Neoplasms, showing its potential as an intervention strategy for obesity-related MASH-Carcinoma of liver (Inoue et al., 2023).

#### 4.5.10 Nobiletin

Nobiletin enhanced the expression of autophagy-related proteins (LC3-II, Beclin1) and key factors of lysosomal function (TFEB, RAB7, LAMP1) in high-fat-diet (HFD) fed ApoE^−/−^ mice by significantly upregulating the expression of these proteins, as well as the expression of free fatty acids (FFA)-treated HepG2 cells autophagy, thereby promoting lipid droplet clearance and alleviating lipotoxic injury (Yang et al., 2022).

#### 4.5.11 Apigenin

Apigenin, a naturally occurring flavonoid compound widely present in fruits and vegetables, has demonstrated significant therapeutic potential in liver‐related diseases such as MASLD. Lu et al. (2020) demonstrated in a palmitic acid‐induced HepG2 cell lipid accumulation model that apigenin restores blocked autophagic flux by downregulating the p‐mTOR/mTOR ratio and reducing P62 expression, thereby promoting autophagic lipid degradation and decreasing intracellular triglyceride and cholesterol content. This mechanism can be blocked by the autophagy inhibitor chloroquine (CQ); Hsu et al. (2021) further demonstrated in oleic acid‐induced hepatocyte models such as Huh7 and HepG2 that apigenin activates the autophagy‐mitochondrial pathway. This mechanism both upregulates autophagy‐associated proteins including Beclin1 and ATG5, thereby promoting the degradation of lipid droplets into free fatty acids, and enhances β‐oxidation‐related proteins such as ACSL1 and CPT1a. Concurrently, it increases the expression of mitochondrial fusion‐associated proteins (OPA1, Mfn2), reduces mitochondrial ROS production, and strengthens mitochondrial network architecture and function. Furthermore, mitochondrial inhibitors (oligomycin, rotenone) attenuate its lipid‐lowering effects. Feng et al. (2017) further revealed an Nrf2‐dependent mechanism in HFD‐induced NAFLD and FFA‐stimulated hepatocyte models. Apigenin directly binds to Nrf2, promoting its nuclear translocation and transcriptional activity, which subsequently inhibits PPARγ‐driven lipogenesis and upregulates antioxidant genes. Nrf2 knockdown/knockout abolished, while Nrf2 overexpression enhanced these effects. This Nrf2‐mediated counteraction of PPARγ activation represents a novel regulatory mode underlying apigenin's amelioration of hepatic steatosis and oxidative stress.

### 4.6 Clinical trial

Flavonoids are natural compounds with broad application prospects; in recent years, their clinical efficacy, drug safety, and practical application outcomes have become hot topics of research in the field. In a prospective cohort study by Bell et al. based on the UK Biobank involving 121,064 adults, participants in the highest quartile of the flavonoid dietary score had a 19% reduced risk of MASLD (HR = 0.81, 95% CI: 0.67–0.97), and both MRI proton density fat fraction (PDFF) and corrected T1 values were significantly improved, providing population-level evidence for the hepatoprotective effects of flavonoids (Bell et al., 2024). A meta-analysis by Li et al., incorporating 12 RCTs, confirmed that flavonoid supplementation significantly reduced levels of ALT, AST, GGT, TG, TC, and LDL-C, inhibited TNF-α and NF-κB-mediated inflammatory pathways, and reduced ultrasound steatosis scores (Li L. et al., 2023).

Randomized controlled trials (RCTs) of flavonoids with different chemical structures have further revealed the heterogeneity of their effects. In a 12-week, randomized, double-blind, placebo-controlled crossover trial involving 41 patients with NAFLD, quercetin at 500 mg/day moderately reduced intrahepatic lipid content measured by MRI-PDFF (from 11.5% ± 6.4%–9.6% ± 5.8%, P = 0.013) and mildly reduced body weight (−1.5 ± 2.6 kg, P < 0.05) and BMI (−0.5 ± 0.9 kg/m^2^, P < 0.05). However, like naringenin and genistein, no significant improvements were observed in ALT, AST, or other secondary outcomes (Li N. et al., 2024). In a 4-week, double-blind RCT, 44 overweight/obese patients with MASLD received naringenin at 200 mg/day. Compared with placebo, naringenin significantly improved steatosis grade, TG, TC, and low-density LDL, and increased HDL (P < 0.05); it also decreased the atherogenic index of plasma (AIP) and visceral fat level. However, no significant changes were observed in ALT, AST or the MASLD fibrosis score (NFS) (Namkhah et al., 2021; Naeini et al., 2022). In an 8-week, randomized, double-blind, placebo-controlled trial, 82 patients with MASLD received genistein at 250 mg/day. Compared with placebo, genistein significantly reduced serum insulin (P = 0.001), HOMA-IR (P = 0.041), MDA (P = 0.004), TNF-α (P = 0.045), and IL-6 (P = 0.018), and decreased waist-to-hip ratio (P = 0.021), body fat percentage (P = 0.015), and TG (P = 0.018). However, similar to naringenin, no significant changes were observed in ALT (P = 0.536) or AST (P = 0.265) (Amanat et al., 2018b). In a 12-week, 1 g/day RCT involving 50 patients, Cheraghpour et al. (Cheraghpour et al., 2019) showed that, compared with placebo, the hesperidin group experienced significant improvements in ALT (P = 0.005), GGT (P = 0.004), TC, TG, and the degree of hepatic steatosis; in parallel, there were significant reductions in the inflammatory markers hs-CRP, TNF-α, and NF-κB, suggesting that hesperidin may exert hepatoprotective effects through inhibition of the NF-κB pathway. In a randomized controlled trial involving 60 patients with MASLD, a 12-week DHM intervention reduced fasting blood glucose, HOMA-IR index, serum LDL-C, and Apo B levels. Apo B levels, while downregulating TNF-α, CK-18 fragment, and FGF21 expression, and upregulating adiponectin levels. It also improved liver function markers including ALT, AST, and GGT, confirming its therapeutic potential for MASLD in clinical settings (Chen et al., 2015).

Flavonoids show remarkable potential in treating the complex pathogenesis of MASLD through the modulation of multiple mechanisms including lipid metabolism, antioxidant, anti-inflammatory, autophagy and gut-liver axis (see Table 1).

**TABLE 1:** Potential bioactive compounds for the treatment of MASLD.

Types	Compound	Level	Study type	Moulding method	Subject	Therapeutic dose	Sample size	Modeling + treatment duration	Positive control	References
Flavones	Breviscapine	B	*In vivo*	HFHC	C57BL/6J	30 mg/kg/d	n = 9	16+8 weeks	—	(Lan et al., 2022)
				MCD	C57BL/6J	30 mg/kg/d	n = 15	8+8 weeks		
				HFD	C57BL/6J	30 mg/kg/d	n = 8	4weeks (simultaneous)		
			*In vitro*	PA + OA	L02	100 μM	n = 4	12h	5Z‐7‐oxozeaenol	
	Nobiletin	B	*In vivo*	HFD	ApoE^−/−^	200 mg/kg/d	n = 16	24 weeks (simultaneous)	—	Yang et al. (2022)
			*In vitro*	FFA	HepG2	40 μg/mL	n = 3	24 h	Chloroquine	
				FFA/DHA	RAW 264.7	20 μg/mL	n = 3	24 h	Chloroquine	
	Baicalin	B	*In vivo*	HFD	C57BL/6J	100 mg/kg/d	n = 9	16+8 weeks	—	Gao et al. (2023)
			*In vitro*	PA	AML‐12	25 μM	n = 3	24 h	Compound C	
	Acacetin	C	*In vivo*	HFD	C57BL/6J	50 mg/kg/d	n = 6−10	16 weeks (simultaneous)	—	Jiang et al. (2023)
			*In vitro*	OA + LPS	HepG2	30 μM	n = 3	24 h	GSK2606414	
	Apigenin	B	*In vitro*	PA	HepG2	40 μM	n = 3	24 h	CQ	Lu et al. (2020);Hsu et al. (2021);Feng et al. (2017)
			*In vitro*	OA	Huh7	40 μM	n = 3	24 h	CQ/BafA1	
			*In vivo*	HFD	C57BL/6J	30 mg/kg/d	n = 9	16 + 3 weeks	Rosiglitazone	
			*In vitro*	FFA	Hepa1‐6	10μM	n = 3	24 h	Sulforaphane	
	Chrysin	C	*In vivo*	HFD	Wistar	75 mg/kg/d	n = 9	3 + 1 week	—	Oriquat et al. (2023)
	Vitexin	C	*In vivo*	HFD	C57BL/6J	20 mg/kg/d	n = 6	8 weeks (simultaneous)	—	Inamdar et al. (2019)
	Wogonin	B	*In vivo*	HFD	C57BL/6J	20 mg/kg/d	n = 12	12 weeks (simultaneous)	—	Chen et al. (2017)
			*In vitro*	PA	NCTC1469	20 μM	n=5	24 h	—	
	Buddleoside	B	*In vivo*	HFHC/HFD	C57BL/7J	30 mg/kg/d	n = 6	12+4 weeks	—	Chen et al. (2025)
				HFD	tfeb‐HKO	30 mg/kg/d	n = 6	12+4 weeks	—	
			*In vitro*	—	HEK293T	40 μM	n = 3	24 h	AICAR/Torin1	
Flavonols	Hyperoside	C	*In vivo*	HFD	Nr4A1^−/−^C57BL/6J	50 mg/kg/d	n = 6−10	16+8 weeks	—	Sun et al. (2021b)
	Quercetin	A	*In vivo*	MCD	C57BL/6J	80 mg/kg/d	n = 6	4weeks (simultaneous)	3‐MA,CQ	Porras et al. (2017), Cao et al. (2023a), Li et al. (2024a)
			*In vitro*	PA	HepG2	100 μM	n = 3	48h	3‐MA,CQ	
			*In vivo*	HFD	C57BL/6J	48 mg/kg/d	n = 10	16 weeks (simultaneous)	—	
			RCT Double-Blind	MASLD	Patients	500 mg/day	n = 41	12 weeks	—	
	Rutin	B	*In vitro*	D‐glucose+μM PA	HeLa	150 μM	n = 3	24 h	Metformin	Liu et al. (2024)
			*In vitro*	OA	HepG2	20 μM	n = 3	24 h	Metformin	
			*In vivo*	HFD	db/db,db/m	200 mg/kg/d	n = 6	8 weeks (simultaneous)	—	
	Kaempferol	B	*In vitro*	OA	HepG2	1 μM	n = 4	24 h	—	(Lu et al., 2022;Liu et al., 2021)
			*In vivo*	HFD	SD	50 mg/kg/d	n = 8	16 weeks (simultaneous)	—	
			*In vivo*	HFD	C57BL/6J	20 mg/kg/d	n = 8	24 + 4 weeks	—	
	Galangin	B	*In vitro*	FFA	HepG2	100 μM	n = 3	24 h	—	Zhang et al. (2020)
			*In vivo*	HFD	C57BL/6J	100 mg/kg/d	n = 4−5	8weeks (simultaneous)	—	
	Fisetin	B	*In vitro*	PA	AML‐12	20 μM	n = 3	24 h	—	Dai et al. (2022)
			*In vivo*	HFD	C57BL/6J	80 mg/kg/d	n = 5−8	16 weeks (simultaneous)	—	
	Myricetin	C	*In vivo*	HFD	Wistar	0.5% (w/w)	n = 8	12 weeks (simultaneous)	—	(Sun et al., 2021b)
Flavanones	Dihydromyricetin	A	*In vivo*	HFD	SD	200 mg kg	n = 6	8+6 weeks	Metformin	Yang et al. (2024), Chen et al. (2015)
			*In vitro*	PA	HEPG2	10 μM	n = 3	24 h	—	
			RCT Double‐Blind	MASLD	Patients	150 mg × 2/day	n = 60	12 weeks	—	
	Naringin	C	*In vivo*	HFD	Wistar	100 mg/kg/d	n = 5	10 + 6 weeks	—	(Sarkar et al., 2025)
	Alpinetin	B	*In vivo*	HFD	C57BL/6J	50 mg/kg/d	n = 8−10	16 + 8 weeks	—	Zhou et al. (2018)
			*In vitro*	Hfru	HL‐7702	80 μM	n =3	24 h	—	
	Taxifolin	B	*In vivo*	HFD	C57BL/6J	3% (w/w)	n = 6	24 + 12 weeks	—	Inoue et al. (2023)
			*In vivo*	WD	Nr4A1^−/−^C57BL/6J	3% (w/w)	n = 11−12	24 + 8 weeks	—	
			*In vitro*	PA	HepG2	10 μM	n = 3	48 h	—	
Isoflavones	Formononetin	B	*In vivo*	HFD	C57BL/6 J	100 mg/kg/d	n = 6	16 weeks (simultaneous)		Wang et al. (2019)
			*In vitro*	FFAs	HepG2	20 μM	n = 3	24 h	Compound C/Chloroquine	
	Puerarin	B	*In vivo*	HCD	C57BL/6J	100 mg/kg/d	n = 8	6 weeks (simultaneous)	—	Fang et al. (2024)
			*In vitro*	0.5 mM FFA	HepG2	80 μM	n = 3	24 h	Pioglitazone	
				LPS + IFN‐γ	RAW 264.7	80 μM	n = 3	24 h	Pioglitazone	
	Genistein	A	*In vivo*	HFD	C57BL/6	32 mg/kg/d	n = 10	22 weeks (simultaneous)	Aspirin	Wang et al. (2018), Amanat et al. (2018b)
			*In vitro*	TNF‐α+ Insulin	HepG2	100 μM	n = 4	24 h	—	
			RCT Double‐Blind	MASLD	patients	250 mg/d	n = 82	8 weeks	—	
	Calycosin	B	*In vivo*	HFD	C57BL/6	50 mg/kg/d	n = 6	12 weeks (simultaneous)	Guggulsterone	Duan et al. (2018)
			*In vitro*	HFFA	L02	10 μM	n = 3	24 h	Guggulsterone	
Chalcones	Licochalcone A	B	*In vivo*	HFD	C57BL/6	10 mg/kg/d	n = 12	16+12 weeks	—	Liou et al. (2019)
			*In vitro*	OA	HepG2	12 μM	n = 3	48 h	ompound C/AICAR	
	Butein	C	*In vivo*	MCD	Wistar	200 mg/kg/d	n = 10	6 weeks (simultaneous)	—	Alshammari et al. (2018)
Flavanes	Hesperetin	A	*In vivo*	HFD	C57BL/6J	50 mg/kg/d	n = 8−10	12 + 6weeks	silymarin	Li et al. (2021);2022b;Cheraghpour et al. (2019)
			*In vivo*	HFD	C57BL/6J	0.2% (wt/wt)	n = 10	16 weeks (simultaneous)	—	
			*In vitro*	OA	HepG2	10 μM	n = 3	24 h	—	
			RCT Double‐Blind	MASLD	Patients	1 g/day	n = 50	12 weeks	—	
	Naringenin	A	*In vivo*	HFD	Wistar	100 mg/kg/d	n = 5	16 + 6 weeks	—	Namkhah et al. (2021);Naeini et al. (2022)
			RCT Double‐Blind	MASLD	overweight/obese Patients	200 mg/d	n = 40	4 weeks	—	
	Bavachin	B	*In vivo*	HFD	C57BL/6	30 mg/kg/d	n = 3−8	20+8 weeks	Rosuvastatin calcium	Wei et al. (2023)
			*In vitro*	OA/Glc and Insulin	AML12/L02/Huh7	20 μM	n = 3	24 h	—	
	Eriocitrin	B	*In vivo*	TAA	C57BL/6	20 mg/kg/d	n = 6	5 + 4 weeks	—	Zhang et al. (2025)
			*In vitro*	TGF‐β	HSC‐T6 and LX‐2	12.5 μM	n = 3	24 h	—	
				LPS, ATP	BMDMs	12.5 μM	n = 3	24 h	—	
	Didymin	B	*In vivo*	HFD	C57BL/6J	0.8 mg/kg/d i.p	n = 8	23 + 3weeks	EX‐527	Yang et al. (2023)
			*In vitro*	PA	AML12	50 μM	n = 3‒4	24 h	EX‐527	
Else	Sappanone A	B	*In vivo*	MCD	C57BL/6	100 mg/kg/d	n = 6	6 weeks (simultaneous)	Silymarin	Zhu et al. (2025)
			*In vitro*	PA	AML12	100 μM	n = 3	24 h	—	
	Trilobatin	C	*In vivo*	HFD	C57BL/6J	60 mg/kg/d	n = 10	18 + 10 weeks	Metformin	Zhang et al. (2022)

### 4.7 Critical appraisal of the included evidence

Evidential strength was systematically assessed for each of the thirty-three flavonoids across four quality dimensions: availability of randomized controlled trial data in MASLD populations, cross-validation in complementary model systems encompassing both dietary and genetic rodent models with multiple hepatocyte lines, mechanistic substantiation via genetic ablation or pharmacological blockade, and adequacy of study design parameters including sample size, intervention duration, and incorporation of positive controls. This evaluation yielded a tiered classification presented in Table 1.

Quercetin, dihydromyricetin, genistein, hesperetin, and naringenin constitute the most robustly supported compounds, each substantiated by at least one double-blind randomized controlled trial alongside multi-model preclinical validation with considerable mechanistic depth. Genistein has been corroborated across high-fat diet-fed rats, MASH rat models, HepG2 cells, and an 8-week clinical trial; quercetin has undergone AMPK-dependent mitophagy dissection via pharmacological blockade with 3-methyladenine and chloroquine, complemented by a 12-week crossover trial. Yet even these compounds exhibit conspicuous evidential gaps. Quercetin’s trial yielded only modest intrahepatic lipid reduction by MRI-proton density fat fraction without transaminase improvement; naringenin’s clinical evidence derives from a brief 4-week pilot study devoid of histological endpoints. No flavonoid has yet undergone large-scale multicenter Phase III evaluation with biopsy-confirmed outcomes—the regulatory benchmark for MASLD pharmacotherapy.

Breviscapine, buddleoside, formononetin, didymin, and kaempferol exemplify a second tier characterized by rigorous target validation through genetic or pharmacological approaches, yet lacking any clinical data. Buddleoside’s engagement of the AMPKβ1-TFEB axis was confirmed via liver-specific *Tfeb* knockout and AMPK inhibition; didymin’s Sirt1 dependency was demonstrated through EX-527 reversal. Nevertheless, their *in vivo* studies employed relatively abbreviated intervention windows with limited group sizes, raising legitimate concerns regarding statistical power and translational relevance to chronic human pathophysiology.

A third stratum—exemplified by chrysin, vitexin, wogonin, and butein—comprises compounds supported solely by single rodent models without mechanistic interrogation extending beyond descriptive pathway-level observations. Several studies within this tier omitted positive controls entirely, and group sizes were frequently minimal, thereby constraining the reliability of effect-size estimation. Hyperoside constitutes an instructive exception: its hepatoprotective effects were completely abrogated in *Nr4a1* knockout mice, convincingly establishing target specificity, though independent replication across alternative dietary models or laboratories remains absent.

Several cross-cutting deficiencies warrant emphasis. The near-exclusive reliance on male C57BL/6J mice limits generalizability given well-established sexual dimorphism in MASLD pathogenesis. Intervention durations rarely exceeded 16 weeks, a period potentially inadequate for capturing fibrosis regression—a clinically meaningful endpoint. Only a minority of investigations employed positive pharmacological comparators, and no head-to-head flavonoid comparison has been conducted. Histological assessment was inconsistently reported; numerous studies relied exclusively on biochemical surrogates and semi-quantitative Oil Red O staining rather than standardized NAS or SAF scoring. The clinical evidence base remains markedly fragmented: collectively, the available randomized controlled trials enrolled fewer than two hundred eighty subjects, employed heterogeneous dosing regimens over disparate durations, and assessed no hard endpoints such as fibrosis stage reversal or MASH resolution.

Conversely, notable methodological strengths warrant acknowledgment. The progressive incorporation of genetic loss-of-function strategies—including *NLRP3* knockout, hepatocyte-specific *Tfeb* deletion, *Sirt3* knockout, and *Nr4a1* knockout models—substantively strengthens causal inference beyond correlative pharmacology. The emergence of multi-omics integration encompassing gut metagenomics and hepatic metabolomics in recent investigations of hesperidin, dihydromyricetin, and rutin represents a methodological advance that, if systematically extended, would markedly elevate evidential quality.

In summation, the flavonoid-MASLD literature manifests a pronounced translational asymmetry wherein mechanistically sophisticated preclinical data contrast sharply with inadequate clinical validation. Priority recommendations include replication of pivotal preclinical findings in female animals and humanized models, standardization of histological endpoints and reporting conventions, execution of adequately powered multicenter randomized controlled trials employing optimized formulations and targeting biopsy-proven MASH resolution, and systematic comparative efficacy assessments to identify the most promising candidates for late-phase development.

## 5 Current challenges and solutions for flavonoids

### 5.1 Current challenges

Flavonoids generally exhibit poor oral bioavailability. According to a summary of human plasma data, the bioaccessibility of quercetin is only about 1.40%, that of cyanidin-3-glucoside is as low as 0.08%, and that of epigallocatechin gallate (EGCG) ranges from 0.16% to 1.78% (Shahidi and Pan, 2022). The factors underlying this bottleneck can be understood at three levels: molecular properties, intestinal disposition, and the food matrix. At the molecular level, Berga et al. (2023) systematically compiled the pKa, logP, and melting point data of flavonoids and pointed out that the vast majority of flavonoid aglycones and glycosides belong to the “brick dust” category—characterized by high melting points and low logP—resulting in poor aqueous solubility and extremely limited dissolution rates, which severely restrict their dissolution and transmembrane transport (Berga et al., 2023). Chemical structure also directly determines their absorption fate: aglycones are absorbed more rapidly than glycosides owing to stronger membrane interactions; compounds with numerous free hydroxyl groups undergo rapid phase II conjugation, leading to low systemic exposure; and pronounced differences exist among subclasses, with isoflavones and flavanones being relatively well absorbed, whereas proanthocyanidins and galloylated catechins are poorly absorbed (Naeem et al., 2022). At the intestinal level, after oral ingestion flavonoids undergo extensive phase II metabolism (glucuronidation, sulfation, methylation) in enterocytes, and some of the resulting metabolites are effluxed back into the intestinal lumen by transporters such as MRPs, creating a futile cycle (Chen et al., 2022; Shahidi and Pan, 2022). The fraction of parent compounds not absorbed in the small intestine reaches the colon, where the gut microbiota converts them into secondary products, almost completely transforming the parent forms (Hu et al., 2025). Williamson et al. (2018) used 14C-labeled epicatechin in a human tracer study to reveal the full metabolic profile: only about 20% of the ingested dose was absorbed in the small intestine as structurally related metabolites, approximately 42% was microbially converted to five-carbon side-chain ring-fission products that were then absorbed, and although total urinary recovery reached 89%, the vast majority of the metabolites differed markedly from the original structure (Williamson et al., 2018). Accordingly, Serreli and Deiana (2019) noted that most current *in vitro* studies still test parent compounds while neglecting metabolic conversion, which represents the fundamental cause of the profound disconnect between *in vitro* activity and *in vivo* efficacy (Serreli and Deiana, 2019). However, low parent-compound bioavailability does not imply that flavonoids lack biological activity *in vivo*. Perez-Vizcaino et al. (2012) illustrated this “flavonoid paradox” using quercetin as a model: quercetin aglycone is virtually undetectable in plasma, yet oral administration produces a clear blood-pressure-lowering effect; its circulating metabolite quercetin-3-glucuronide can be hydrolyzed by local β-glucuronidase in the vascular wall, releasing the active aglycone, which then accumulates in tissues. Inhibition of this enzyme completely abolished the antihypertensive effect of orally administered quercetin, confirming that a “conjugation-deconjugation cycle” is an essential prerequisite for its *in vivo* activity (Perez-Vizcaino et al., 2012). Oteiza et al. (2018) further noted that flavonoids can reach millimolar concentrations in the gastrointestinal lumen (far higher than the nanomolar levels in the systemic circulation) and can exert indirect systemic effects through local actions such as protecting intestinal barrier integrity and modulating gut hormone secretion—for instance, non-absorbable proanthocyanidins can still attenuate obesity in high-fat, high-fructose-fed mice (Oteiza et al., 2018). Thus, as suggested by Williamson et al. (2018), the core challenge in flavonoid bioavailability research is not merely to “elevate plasma concentrations,” but rather to understand the biological efficacy of different chemical forms (parent compounds, conjugated metabolites, and microbial metabolites) at their respective sites of action (Williamson et al., 2018). At the food matrix level, flavonoids are almost never ingested as pure compounds; instead, they coexist with lipids, proteins, carbohydrates, and minerals, forming the food matrix, and the complex physicochemical interactions among these metabolites profoundly affect their release and absorption (Zhang J. et al., 2024). Free fatty acids generated during lipid digestion can solubilize hydrophobic flavonoids and participate in mixed-micelle formation, thereby positively promoting absorption, although excessive lipids may hinder sustained release. The influence of proteins depends strongly on their amino acid composition and the flavonoid structure: β-lactoglobulin increased the bioaccessibility of EGCG, epigallocatechin (EGC), and epicatechin (EC) by 52.6%, 85.0%, and 37.0%, respectively, whereas β-casein exerted negative effects on EGCG and EGC; consumption of blueberries with milk decreased the peak plasma concentrations of caffeic acid and ferulic acid in subjects by 49.7% and 19.8%, respectively. Dietary fiber adsorbs flavonoids via hydrogen bonding, which can reduce their absorption in the small intestine and deliver them to the colon Moreover, the ortho-dihydroxy phenolic groups of flavonoids can chelate minerals such as non-heme iron; this is regarded as an “antinutritional” effect, but it may also alter the interaction pattern between flavonoids and cell membranes. Food processing methods are likewise not negligible—heat treatment can reduce total flavonoid content by 12%–25%, and sun drying also negatively affects bioaccessibility (Shahidi and Pan, 2022; Zhang J. et al., 2024).

### 5.2 Solutions for flavonoids

Enhancing the bioavailability of flavonoids constitutes a critical prerequisite for successfully translating their pharmacological activities into clinical applications. These compounds universally face significant challenges, including poor water solubility, rapid phase II metabolism (notably glucuronidation and sulfation), and consequently low oral bioavailability. Take hesperidin as an example; it exhibits extremely low water solubility (<0.01%) and poor oral bioavailability due to its rutinoside moiety. In contrast, its aglycone form, hesperetin, demonstrates significantly superior bioavailability owing to the absence of this sugar group (Sivaslioglu and Goktas, 2025). Similarly, quercetin, isoquercitrin, and rutin, despite sharing the identical flavonoid core, exhibit vastly different absorption kinetics and bioavailability profiles due to the distinct glycosidic substitutions (aglycone, monoglucoside, and rutinoside, respectively). The monoglucoside derivative, isoquercitrin, can be efficiently hydrolyzed by intestinal β-glucosidase into quercetin for rapid absorption, exhibiting notably higher bioavailability than both quercetin (aglycone) itself and the diglycoside rutin (Shi et al., 2022).

To overcome these inherent limitations, current research focuses on two primary optimization strategies:For instance, chrysin exhibits an oral bioavailability of less than 1%, primarily hindered by extensive Phase II metabolism at its hydrophilic 7-hydroxyl group and intrinsic low solubility. To address this, Zhang et al. (Zhang R. et al., 2024) introduced a hydrophilic carbamate-based promoiety onto this metabolically active seven-position, successfully masking the metabolic site and enhancing solubility. The synthesized derivative, C-1, achieved an oral bioavailability of 24.22% in db/db diabetic mice model and demonstrated significantly improved efficacy over native chrysin in ameliorating MASLD.

The absolute oral bioavailability of baicalin is only about 2.2%. To improve its absorption, Liu et al. (Liu et al., 2020) successfully encapsulated it into nanoliposomes (BA-NL). In MCD diet-induced murine MASLD models, BA-NL displayed substantially enhanced pharmacological effects compared to free baicalin, including superior inhibition of hepatic lipid accumulation, mitigation of liver fibrosis, and more effective suppression of the TLR4 inflammatory signaling pathway. Similarly, dihydromyricetin (DMY) suffers from low (∼4%) oral bioavailability. Yin et al. (Yin et al., 2026) developed a bioinspired nano emulsion (DMY-bNE) with high drug loading (24.9%) and encapsulation efficiency (99.5%) via a double-emulsion method combined with sodium cholate decoration. This system markedly enhanced the gastrointestinal absorption and liver-targeted accumulation of DMY, thereby effectively mitigating MASLD progression through robust inhibition of hepatocyte ferroptosis. Furthermore, delivery studies on luteolin and kaempferol highlight the considerable promise of nanotechnology: Liu et al. (Liu et al., 2022) encapsulated luteolin within mPEG-PLGA nanoparticles coated with Eudragit S100 enteric polymer, achieving targeted colonic delivery; Ahmed et al. (Ahmed et al., 2022) loaded luteolin into zinc oxide nanoparticles (Lut/ZnO NPs), enhancing its solubility and bioavailability and demonstrating its efficacy in ameliorating MASLD-associated insulin resistance via modulation of the PI3K/AKT/FoxO1 pathway *in vivo*; Oanh et al. (Oanh et al., 2023) developed innovative nanoparticles co-encapsulating astaxanthin and kaempferol, significantly improving kaempferol’s water solubility and stability, and observed synergistic suppression of oxidative stress and lipid accumulation in HepG2 cell models.

## 6 Conclusion

Metabolic dysfunction-associated steatotic liver disease (MASLD) (MASLD) has emerged as the most prevalent chronic liver disorder globally and a leading contributor to morbidity and mortality, imposing a substantial burden on healthcare systems worldwide. Despite its growing impact, the pathogenesis of MASLD remains incompletely understood, and no pharmacological agents have been specifically approved for its prevention or treatment. Flavonoids, as natural bioactive compounds, present a promising therapeutic alternative due to their multifaceted biological activities and favorable safety profiles. They exert their effects through multiple mechanisms, including modulating lipid metabolism by targeting SREBP-1c and PPARs, alleviating oxidative stress via the Nrf2 pathway, suppressing inflammatory responses by inhibiting NF-κB and NLRP3 inflammasome activation, and improving insulin resistance by activating the PI3K/Akt signaling pathway. However, the clinical application of flavonoids is currently limited by their low bioavailability. To address this challenge, innovative strategies such as nanotechnology-based delivery systems should be explored to enhance their bioavailability and therapeutic efficacy in MASLD.

Moreover, there is a pressing need for further research to determine which specific flavonoids are the most effective and suitable for dietary interventions. While preclinical studies have provided valuable insights into the potential of flavonoids, their efficacy and safety in humans require rigorous validation through well-designed clinical trials. In this review, we have comprehensively summarized the classification, biochemical properties, and biological functions of flavonoids, as well as their molecular targets and mechanisms in MASLD. Our aim is to highlight the pharmacological potential of flavonoids and provide a scientific foundation for the development of novel therapeutic strategies based on natural products. By advancing our understanding of flavonoids, we hope to contribute to the prevention and treatment of MASLD and improve the outcomes for patients affected by this complex metabolic liver disease.

## Glossary

AAAs: AromaticAminoAcids
AhR: ArylHydrocarbonReceptor
AMPK: Adenosine monophosphate-activated protein kinase
AP: Autophagy-relatedProtein
ATG: Autophagy-relatedGene
BCAAs: Branched-chainAminoAcids
BA: BileAcid
BAs: BileAcids
CPT1α: CarnitinePalmitoyltransferase1α
CHOP: C/EBPHomologousProtein
COX-1: Cyclooxygenase-1
ER: EndoplasmicReticulum
FASN: FattyAcidSynthase
FFA: FreeFattyAcid
FGFR: FibroblastGrowthFactorReceptor
FXR: FarnesoidXReceptor
G6Pase: Glucose-6-phosphatase
GPRs: G-proteinCoupledReceptors
GPX4: GlutathionePeroxidase4
HCC: HepatocellularCarcinoma
HFD: High-fatDiet
HSC: HepaticStellateCell
HSP: HeatShockProtein
IGF-1: Insulin-likeGrowthFactor1
IKKβ: IκBKinaseβ
IL-6: Interleukin-6
IL-1β: Interleukin-1β
IR: InsulinResistance
JNK: c-junN-terminalKinase
Keap1: Kelch-likeECH-associatedProtein1
LBP: Lipopolysaccharide-bindingProtein
LPS: Lipopolysaccharide
MAMPs: Microbial-associatedMolecularPatterns
mTOR: MammalianTargetofRapamycin
NAFL: Non-alcoholicFattyLiver
MASLD: Non-alcoholicFattyLiverDisease
**MASH**: Non-alcoholicSteatohepatitis
NF-κB: NuclearFactorKappa-B
NLRP3: NOD-likeReceptorFamilyPyrinDomainContaining3
Nrf2: NuclearFactorErythroid2-relatedFactor2
PI3K: Phosphatidylinositol3-kinase
PPAR: PeroxisomeProliferator-activatedReceptor
RAGE: ReceptorforAdvancedGlycationEndproducts
ROS: ReactiveOxygenSpecies
SCD1: Stearoyl-CoADesaturase1
SCFAs: Short-chainFattyAcids
SREBP-1c: SterolRegulatoryElement-bindingProtein1c
T2DM: Type2DiabetesMellitus
TGF-β: TransformingGrowthFactorβ
TLR: Toll-likeReceptor
TNF-α: TumorNecrosisFactorα
TXA2: ThromboxaneA2
UCP1: UncouplingProtein1
VLDL: VeryLow-densityLipoprotein
